# Comorbidity of adult ADHD and substance use disorder in a sample of inpatients bipolar disorder in Iran

**DOI:** 10.1186/s12888-022-04124-6

**Published:** 2022-07-19

**Authors:** Rahim Badrfam, Atefeh Zandifar, Mahdi Barkhori Mehni, Malihe Farid, Fatemeh Rahiminejad

**Affiliations:** 1 Department of Psychiatry, Faculty of Medicine, Roozbeh Hospital, Tehran University of Medical Sciences, Tehran, Iran; 2 Social Determinants of Health Research Center, Alborz University of Medical Sciences, Karaj, Iran; 3 Department of Psychiatry, Imam Hossein Hospital, Alborz University of Medical Sciences, Karaj, Iran; 4 Faculty of Medicine, Tehran University of Medical Sciences, Tehran, Iran; 5 Non Communicable Diseases Research Center, Alborz University of Medical Sciences, Karaj, Iran

**Keywords:** Bipolar disorder, Adult ADHD, Substance use disorder, Comorbidity

## Abstract

**Backgrounds:**

The study of the relationship between adult Attention deficit hyperactivity disorder (ADHD) and bipolar disorder has received more attention in recent years and there is limited information in this area. On the other hand, there is a significant comorbidity between ADHD and bipolar disorder with substance use disorder. In this study, we investigated the prevalence of comorbidity of adult ADHD and substance use disorder among a group of bipolar patients admitted to a psychiatric hospital.

**Methods:**

One hundred fifty patients from a total of 200 consecutive patients who were referred to the emergency department of Roozbeh Psychiatric Hospital in Tehran, diagnosed with bipolar disorder based on the initial psychiatric interview and needed hospitalization, were evaluated again by an experienced faculty member psychiatrist by using a subsequent interview based on the Diagnostic and Statistical Manual of Mental Disorders, 5th Edition(DSM-5).

They were evaluated using the Structured Clinical Interview for DSM-5 (SCID-5) questionnaire to confirm the diagnosis of bipolar disorder and the comorbidity of adult ADHD and substance use disorder.

**Results:**

From 150 patients diagnosed with bipolar disorder, 106 patients (70.7%) had adult ADHD. 89 patients (59.3%) had substance use disorder and 58 patients (38.7%) had both of these comorbidities with bipolar disorder. Comorbidity of adult ADHD was associated with the earlier onset of the first mood episode in bipolar disorder (*p* value = 0.025). There was no statistically significant relationship between substance use disorder and age of onset of the first episode. (*P* value = 0.57).

**Conclusions:**

Due to the limitations of studies on adult ADHD comorbidity with bipolar disorder, especially in hospital settings, as well as the increased risk of association with substance use disorder, further multicenter studies in this area with larger sample sizes can increase awareness in this regard.

## Background

Although the global mental health survey estimates the prevalence of lifelong and 12-month bipolar disorder at 2.4 percent and 1.5 percent, respectively, there are many differences between countries that may be due to methodological or even cultural differences [1]. However, up to one-third of cases of bipolar disorder are not recognized until about ten years after the onset of the disease [2]. They spend a significant part of their lives with symptoms of depression or mania [3, 4].

Early onset of bipolar disorder is reported to be associated with a lower prognosis, longer treatment delays, and more association with substance use disorders [5]. Early and accurate diagnosis of this disorder is challenging in clinical practice due to the presence of nonspecific symptoms and more mood lability and the greater prevalence of depressive episodes at the onset of the disease [6].

On the other hand, with the increasing recognition of attention deficit hyperactivity disorder (ADHD) in adults and psychotic disorders in children and adolescents, the possibility of a link between ADHD and bipolar disorder has been raised more than ever [7]. However, there is limited information on the prevalence of adult ADHD and its correlations [8]. It is estimated that its prevalence in the general population in different communities is between 1.2 to 7.3% [9].

Despite various studies, the exact association between ADHD and bipolar disorder remains unclear in many respects [10]. The results of many of these studies show a significant overlap between the symptoms of these two disorders in some respects [11–13]. The high level of this overlap of clinical symptoms, such as excessive talking and increased motor activity seen in all age groups has further emphasized the possibility of such an association [14]. As some studies show, the comorbidity of these two disorders seems much higher than expected [15]. Also, findings from genetic, neurochemical, and neuroimaging studies, along with clinical and epidemiological findings, indicate a possible association between the closeness of biological findings and clinical manifestations between the two disorders [16, 17]. In addition to high comorbidity among the symptoms of these two disorders, their pathophysiological proximity has also been considered [18].

There are various reports of a higher prevalence of ADHD among patients with bipolar disorder than in the general population [19] This issue is especially important in the age range of children and adolescents, so that some reports mention ADHD comorbidity among children with bipolar disorder up to 85% and the prevalence of comorbidity of bipolar disorder among patients with ADHD up to 22% [15]. However, there are different views in this field and attention to different aspects related to the epidemiology, biology and psychoanalysis of these disorders has been emphasized in this field [20]. For example, in a recent meta-analysis, it was reported that up to one in 13 patients with ADHD have comorbidity with BD and up to one in six patients with BD have comorbidity with ADHD [21]. Also, according to some references, adults with ADHD and comorbid BD have more severe clinical manifestations, lower quality of life, higher number of mood episodes, and higher prevalence of substance use [22].

On the other hand, the role of comorbidities associated with ADHD is important because of the long-term or lifelong nature of some of these disorders [23]. Some studies have identified early-onset bipolar disorder as a subset of the disorder which is associated with a greater genetic predisposition to other psychiatric disorders, and thus suggests that it is more likely to be associated with disorders such as ADHD [11, 15, 24]. According to the results of a large study in this field, in addition to the genetic correlation between these two disorders, we can consider the potentially different genetic mechanisms in the formation of the time of onset of bipolar disorder [25]. At the same time, some studies emphasize the lack of sufficient evidence for cross-transmission between bipolar disorder and ADHD among their first-degree relatives. Thus, such a correlation between the two disorders was attributed to items such as a diagnostic artifact or the presence of specific subtypes of bipolar disorder [26]. However, in a recent analysis of the results of large genome-wide association studies, a report on shared underlying genetic risk for ADHD and bipolar disorder was presented [27].

ADHD, meanwhile, is a common comorbid disorder with substance use disorder with a worse prognosis [28]. Various studies have emphasized the role of different gene types along with environmental risk factors in the formation of substance dependence [29]. Another important point about patients with bipolar disorder is the high co-occurrence of bipolar disorder with substance use [30, 31]. The genetic, neurobiological, and neuropsychological relationship between ADHD and alcohol use/ alcohol use disorder has also been suggested in some studies, and for this reason, the need for routine screening for ADHD among patients with alcohol use disorder has been emphasized [32]. Another study highlighted the common genetic background between ADHD and substance use disorder and the causal effect of ADHD on this disorder [33]. Also, the comorbidity of substance use disorder at the time of ADHD diagnosis, according to some studies, has been associated with more cognitive deficits and poorer functional outcomes [34] The high prevalence of this comorbidity, along with other related comorbidities, has made patients with bipolar disorder more prone to other mental health disorders [35].

The earlier age of onset of bipolar disorder, due to its greater association with comorbidities and poorer prognosis, indicates the need for earlier diagnosis of the disorder and appropriate pharmacological and psychological therapeutic measures [5].

Due to the limited studies conducted on the co-occurrence of adult ADHD and bipolar disorder and the need to achieve the diagnostic validity of these comorbidities on the one hand and the need to simultaneously pay attention to the comorbidity of substance use disorder with the above two disorders, in this study we have evaluated these important comorbidities among a group of patients with bipolar disorder admitted to a referral psychiatric hospital.

The relationship between adult ADHD and bipolar disorder has been seriously considered by experts in recent years, and it seems necessary to fill the knowledge gap by conducting studies in different hospital / clinic settings and / or referral conditions. On the other hand, the comorbidity between bipolar disorder and substance use disorder, as one of the most common psychiatric comorbidities, has long been considered [36]. The main purpose of this study was to investigate the prevalence of comorbidity between these three important psychiatric disorders in hospital setting and among patients who in many cases have been referred from other diagnostic / therapeutic centers.

Trying to gain more insight into the pathophysiology of bipolar disorder associated with adult ADHD and substance use disorders, given the new therapeutic aspects involved and how to proceed with subsequent health care, can be the next steps in this evaluation [37].

## Material and method

### Sample size

In order to achieve the appropriate sample size, using the sample size formula in the study of the prevalence of the disorders (the following formula), we obtained a relative estimate of the required sample size.

$$n = \;\frac{{z_{1 - a/2}^{2} \; \times \;p\left( {1 - p} \right)}}{{d^{2} }}$$

In this formula, “n” is the number of participants in the study, “p” is the estimation of the ratio in the study population (assuming about 50% comorbidity of substance use disorder and ADHD among patients with bipolar disorder referred to the emergency department of Roozbeh Hospital), “d” is the accuracy of estimating the prevalence and α is the estimation of the first type error.

According to the estimated prevalence of 0.5 of comorbidity of substance use disorder and ADHD among patients with bipolar disorder (*p* = 0.5) who were referred to the emergency department of Roozbeh Hospital, the accuracy of estimating the prevalence of 0.1 (d = 0.1) and the first type error of 0.05 (α = 0/05), the sample size required to estimate the prevalence of bipolar disorder in this study was 96 patients. Finally, a total of 200 consecutive patients were considered as the sample size of the study.”

### Study design

One hundred fifty patients from a total of 200 consecutive patients who were referred to the emergency department of Roozbeh Psychiatric Hospital in Tehran, diagnosed with bipolar disorder based on the initial psychiatric interview and needed hospitalization, were evaluated again by an experienced faculty member psychiatrist by using a subsequent interview based on the Diagnostic and Statistical Manual of Mental Disorders, 5th Edition(DSM-5). They were evaluated using the Structured Clinical Interview for DSM-5 (SCID-5) questionnaire to confirm the diagnosis of bipolar disorder and the comorbidity of adult ADHD and substance use disorder.

Also, for evaluation in terms of recent substance use, patients underwent urinalysis.

Using the researcher-made questionnaire, other information such as demographic information, psychiatric history, etc. were obtained from the patient and her/his family. If any of the patients wanted to leave the study, they could leave it at any stage.

This study was conducted between November 2019 and February 2020. Data collection in this study was done by one of the senior psychiatry residents of Roozbeh Hospital together with one of the psychiatry interns (a medical student), under the direct supervision of a faculty member of the department of psychiatry of Tehran University of Medical Sciences. The various stages of data collection and analysis have also been performed under the direct supervision and execution of one of the statistical experts.

### Inclusion and exclusion criteria

All patients over the age of 18 referred to the psychiatric emergency department, who had admitted to hospital due to their psychiatric status after the initial assessment and had been diagnosed with bipolar disorder, entered to the study with informed consent.

Exclusion criteria included the patient's unwillingness to continue attending the study at any stage of the study, as well as the patient's leaving the hospital for any reason, before completing the desired assessments.

### Procedure

#### The site

The emergency department and other hospital wards of Roozbeh Psychiatric Hospital in Tehran were the study sites. This hospital has 16 emergency beds and 220 beds in different wards. It is the largest academic psychiatric hospital in Iran and many of its patients are complex psychiatric cases who are referred from different hospital centers or other cities in Iran for diagnostic and therapeutic measures.

In addition to inpatient wards, this hospital has a daily outpatient clinic for continuous outpatient care of patients discharged from the hospital as well as for other outpatient visits. The hospital also has a separate inpatient ward and outpatient clinic for the treatment of patients with substance use-related problems (in addition to patients with comorbid disorders with substance use disorder). The subspecialty ward of children and adolescents and the related outpatient clinic along with the occupational therapy and therapeutic exercise units and daily center for the care of patients with severe psychiatric disorders are other wards of this psychiatric hospital.

In this center, patients with a variety of psychiatric disorders, especially patients with more severe symptoms and comorbidity with other psychiatric disorders and referrals with diagnostic and therapeutic complexities, receive medical and diagnostic care.

The average number of annual visits in the emergency department of this hospital is more than 5000 visits. Trained psych-nurses are responsible for caring for patients without the need for the presence of companions. In a limited way and in necessary circumstances, court-mandated admissions are done in this center. The typical length of stay is between two weeks to one month, which is tried to be reduced to a minimum by expanding the support services such as social work services, providing various types of psychiatric and psychological care services and equipping the day center.

This hospital is an educational and therapeutic academic center which is affiliated with Tehran University of Medical Sciences and the services provided in it are covered by various medical insurances. Patients and their families pay a limited fee for inpatient services or outpatient visits.

#### Selection of participants

Patients were included in the study based on the available sampling method and consecutive selection of patients based on the mentioned criteria and after obtaining written consent. Interviews and completion of relevant forms and the use of study tools were done after stabilization of the psychiatric conditions of patients in the emergency department or after transfer to the inpatient wards. In particular, the diagnostic evaluation for adult ADHD was performed at another time point after recovery from the acute episode.

Also, especially in patients with acute manic / mixed episodes, special attention was paid to the symptom count level versus the diagnostic level in order to increase the accuracy in identifying the symptoms related to ADHD comorbidity. Regarding the patients in the mania episode, special attention was paid to the psychiatric history of the patients based on the history taken from the patients' families. Due to the severity of symptoms and short duration of initial symptoms in patients in this psychiatric referral center, in all cases, the interval between the onset of symptoms and the time of admission was less than one month before admission. Accordingly, in addition to examining patients in the last stages of hospitalization before discharge, the condition of patients in longer periods before hospitalization (including 6 months before recent hospitalization) was carefully considered and evaluated by an experienced psychiatrist and faculty member.

In this situation, special attention was paid to the symptoms related to psychomotor activity, speech and concentration. The above symptoms were considered as part of the ADHD criteria if, in addition to the comorbidity of the symptoms in the recent episode, they were associated with a decline in social, educational or occupational function outside of the recent episode, according to the history taken. Also, focusing on the Diagnostic and Statistical Manual of mental disorders-fifth edition(DSM-5) diagnostic criteria, symptoms were considered ADHD symptoms only if they did not occur exclusively during the recent episode and were not better justified by other associated problems such as substance withdrawal or intoxication.

#### Scale

### Structured clinical interview for the diagnostic and statistical manual of mental disorders-fifth edition(DSM-5) (SCID-5)

SCID-5 is a valuable tool for use in clinical settings. Interview questions rate each criterion, whether or not they exist, according to SCID-5 diagnostic criteria [38]. Osorio et al., in their assessment with 12 psychiatrists / psychologists present as raters and observers, reported a positive agreement between interview and clinical diagnoses ranging from 73 to 97% [39]. They also reported diagnostic sensitivity / specificity greater than 0.70. Also, in the joint interview, kappa levels for most diagnoses were reported > 0.70 and positive agreement > 75%.

Mohammadkhani et al., have evaluated psychometric properties of Persian translation of this tool in terms of reliability and validity [40]. They announced psychometric properties of this instrument, by examining clinical and non-clinical cases, in the acceptable range in terms of internal consistency, test–retest reliability and Kappa reliability as (0.95—0.99), (0.60—0.79) and (0.57—0.72), respectively.

In this study, SCID-5 was used to diagnose ADHD. Somma et al., in their study, emphasized the reliability and validity of this tool for this purpose. In their report, they mentioned the general omega coefficient value 0.88. They also stated that general ADHD factor explained 81.0% of the SCID-5 ADHD criteria reliable score variance [41]. Also in a study in Iran, diagnostic agreement was 80% between SCID-5 and Conner’s Adult ADHD Rating Scale-Self Report-Screening Version (CAARS-S-SV) [42].

### Statistical methods

Statistical analysis was performed using SPSS v21. Continuous variables were summarized as mean (SD) and categorical variables as proportions (%). The relationship between qualitative characteristics was test using χ2 Test and Fisher's Exact Test. *P* values of < 0.05 were considered to be statistically significant.

### Ethical considerations

This study was performed based on the code of ethics obtained from the ethics committee of Tehran University of Medical Sciences with the code number IR.TUMS.MEDICINE.REC.1399. 421. All participants and their caregivers were aware of the study and how it was conducted when they entered the study. All the provisions of the Helsinki Declaration were taken into account in the implementation of this study. Study participants were assured that all information obtained from the study was confidential. Written informed consent was received from all the study participants and their caregivers.

## Result

The total number of participants in the study included 150 patients with bipolar disorder referred to the emergency department of Roozbeh Hospital in Tehran who had been admitted to the emergency room. 69 (46%) of them were men and 81 (54%) were women. The mean age of study participants was 35.1 (standard deviation = 11.9) years. 65 of them (43.3%) were single, 57 (38.0%) were married, 24 (16%) were divorced and 4 (2.7%) were widows. Below diploma education with 67 patients (44.0%) had the highest prevalence in terms of level of education among participants. Other demographic information is provided in Table 1.

**Table 1:** Demographic status of patients diagnosed with bipolar disorder in the study

Socio-Demographic Characteristics		N(%)
Gender	Male	69(46.0)
	Female	81(54.0)
Mean age	-	35.1(year)(SD*=11.91)
Marital status	Single	65(43.3)
	Married	57(38.0)
	Divorced	24(16.0)
	Widowed	4(2.7)
Education	Illiterate	4(2.7)
	Below diploma	67(44.7)
	Diploma	53(35.3)
	Bachelor	24(16)
	MSc or higher	2(1.4)
Occupational status at the time of hospitalization	Unemployed	45(30.0)
	Worker	15(10.0)
	Eemployee	11(7.3)
	Housewife	62(41.3)
	Labourer	14(9.3)
	Student	3(2.0)
Having child	Yes	68(45.3)
	No	82(54.7)

According to DSM-5 criteria, 64 (42.7%) were in the depressed episode and 86 (57.3%) were in the manic episode. 20(3.13%) patients were admitted for the first time and the rest had a previous history of psychiatric hospitalization.

**Fig. 1:**
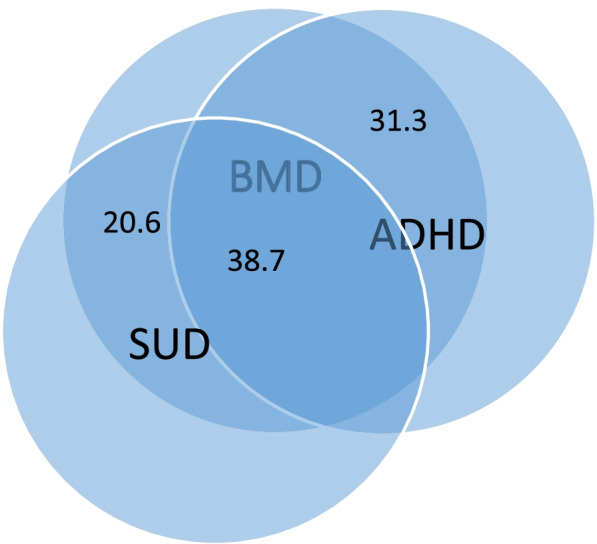
Overlap between Bipolar Mood Disorder (BMD), Attention Deficit Hyperactivity Disorder (ADHD) and Substance Use Disorder (SUD) in patients under study

One hundred six patients (70.7%) had adult ADHD. 89 patients (59.3%) had substance use disorder. 58 (38.7%) patients had both adult ADHD and substance use disorder comorbidity. (Fig. 1) 72(48%) patients with bipolar disorder had a recent history (last two weeks) of substance use. All patients with a recent history of substance use met the criteria for substance use disorder. The combination of amphetamine and opium use was the highest use among them (36 patients (50%))0.17 (23.6%) patients also had a recent history of amphetamine use alone. Opium and cannabis use were in 11 patients (15.3%) and 8 patients (11.1%), respectively (Table 2).

**Table 2:** Psychiatric history of patients with bipolar disorderin the study (ADHD: Attention Deficit Hyperactivity Disorder)

Psychiatric history of patients		N(%)
Age at onset of the first episode	< 18year old	86(57.3)
	18-25year old	45(30)
	< 25year old	19(12.7)
Substance use disorder	positive	89(59.3)
	negative	61(40.7)
Substances use at the same time with the recent episode (one recent month)	Amphetamine	17(23.6)
	Opium	11(15.3)
	Cannabis	8(11.1)
	Combination of amphetamine and opium	36(50.0)
	Total	89(100)
Comorbidity of history of Adult ADHD and bipolar disorder	Positive	106(70.7)
	Negative	44(29.3)

Comorbidity of ADHD was statistically associated with the earlier onset of the first episode in bipolar patients (*p* value = 0.025 based on Fisher's exact test). In patients with bipolar disorder with the first episode under the age of eighteen, 68 patients out of 86 (79.1%), in the age group of 18 to 25 years, 30 out of 45 (66.7%) and in the age group over 25 years, 8 out of 19 (42.1%) and in total, 106 out of 147 patients (70.7%) had comorbidity with Adult ADHD.

There was no statistically significant relationship between substance use and the age of onset of the first episode (*P* value = 0.57 based on Fisher's exact test). In patients with bipolar disorder under study, in the age group under 18 years, 50 patients out of 86 (58.1%), in the age group of 18 to 25 years, 26 patients out of 45 (57.8%), in the age group over 25 years, 13 patients out of 19 (68/4%) and a total of 89 patients out of 150 (59.3%) had substance use disorder.

There was no statistically significant relationship between comorbidity of ADHD in patients with bipolar disorder referred to the emergency department of Roozbeh Hospital and substance use disorder in them (*p* value = 0.07 based on Chi-square test). Out of 106 patients with bipolar disorder with comorbidity of Adult ADHD, 58 patients (54.7%) and out of 44 patients with bipolar disorder without comorbidity of Adult ADHD,31 patients (70.5%) had comorbidity of substance use disorder.

## Discussion

In this study, conducted at an academic referral psychiatric hospital in Iran, we found a high prevalence of adult ADHD and substance use comorbidities among patients with bipolar disorder. There was a statistically significant relationship between adult ADHD and early onset of bipolar disorder (under 18 years). However, there was no statistically significant relationship between substance use in bipolar patients and the age of onset of the disorder. Also, there was no statistically significant relationship between adult ADHD in bipolar patients and substance use.

Attention to the comorbidity of these three disorders is especially important because in many studies, only limited comorbidities between some of these disorders are addressed. Also, the existence of these complex comorbidities is often associated with diagnostic complications and therapeutic resistance. This is especially reported in cases requiring hospitalization in referral centers.

In a study of 56 patients with bipolar disorder, Sachs et al. examined the possible association between childhood ADHD and the early onset of bipolar disorder (the onset of the first episode under the age of 19) [10]. They reported a higher prevalence of early-onset bipolar disorder in patients with ADHD comorbidity compared with those without the disorder. The results of our study are similar to the results of this study. In our study, there was a statistically significant relationship between the symptoms of ADHD in adulthood, which can indicate childhood disorder [43], and the early-onset of bipolar disorder. In our study, with a high prevalence of adult ADHD among participants, 57.3% of them experienced their first episode of bipolar disorder under the age of 18. It seems that the presence of early mood disorders in people with a history of ADHD should be given more attention in terms of the risk of bipolar disorder [44].

In a study in Italy, Masi et al. surveyed 59 patients with bipolar disorder under the age of 18 and reported that about a quarter of them had ADHD comorbidity at the onset of the first episode [45]. They also emphasized the association between ADHD and the onset of the first bipolar episode at a younger age and described the presence of ADHD comorbidity in bipolar patients as common and stressed the need to pay attention to it. Given the onset of ADHD in childhood, it appears that a large proportion of the patients in our study also had ADHD symptoms during childhood. In this respect, the results of our study are close to the results of the recent study, although differences in the possible prevalence of this comorbidity in childhood may be related to differences in measurement tools. In addition, the high prevalence of adult ADHD in our study among patients with bipolar disorder could point to other aspects such as biological differences [46, 47]. Another important point in this regard is to pay attention to cultural differences in the formation of diagnostic processes. In this regard, paying attention to the degree of deviance from cultural norms is very essential. This is a point that in newer editions of the Diagnostic and Statistical Manual of Mental Disorders (DSM) has also received special attention. Issues such as differences in language norms, religion, beliefs, and gender roles are influential points in this regard [21].

Attention to inter-individual differences and variations in the context of the development of inattention symptoms is important in this regard. In general, different sets of genes have been reported as important and influential factors at the baseline and developmental coarse of ADHD symptoms [48].

Also, paying attention to the impact of environmental factors on the course of the disease and the role of treatment and care measures in this field can be effective in forming the transfer of ADHD from one age period of life to another. In this regard, more emphasis is placed on the impact of environmental factors on neurodevelopmental processes. A point that can be changed to some extent with social context [49]. In this context, the need for related studies, focusing on this topic, can be considered. Due to the relatively high prevalence of psychiatric disorders in children and adolescents in Iran [50, 51] and also the unfavorable conditions associated with child and adolescent abuse in Iran based on some important studies [52], some of these conditions may be considered effective in aggravating these symptoms.

On the other hand, Duffy, in a review study, stated that the clinical diagnosis of ADHD in children of patients with bipolar disorder is not a reliable predictor of bipolar disorder. However, he reported symptoms of anxiety and depression, as well as inattention among these children and adolescents, as part of the evolve of bipolar disorder based on some evidence. Also, in their study, the risk of ADHD was not increased among the children of patients with bipolar disorder [53]. In our study, it can be stated that the high prevalence of comorbidity between ADHD and bipolar disorder and the significant relationship between this comorbidity and the age of onset of bipolar disorder in the population under study, may indicate the need to pay attention to symptoms of anxiety, depression, severe irritability or severe mood swings in childhood and adolescence, especially in patients with a family history of mood disorders, as possible early symptoms of bipolar disorder [54–56]. This is especially important because some studies have placed great emphasis on the biological aspects of bipolar disorder [57]. However, in this context, other factors related to this situation, such as substance use, should also be considered [58].

Wingo et al., in a systematic review, noted an increasing diagnosis of adult ADHD and highlighted the small number of studies related to comorbidity of adult ADHD and bipolar disorder [59]. They described this comorbidity as fairly common based on studies and stressed the need to continue related studies to identify the true prevalence of this comorbidity. They also reported comorbidities in up to 47% of patients with adult ADHD and 21% of patients with bipolar disorder, and stressed the more severe course of the disease and the greater number of comorbidities in these conditions. They also emphasized the need to mood stabilization in these situations.

However, various studies report different prevalence of adult ADHD among patients with substance use disorders. According to some studies, the prevalence of adult ADHD in this group of patients is estimated at 13% [60]. As the prevalence of adult ADHD in the general population in the study of Kessler et al. was estimated to be 4.4% [8]. Based on the results of their study, they stated that many of these patients are not identified and treated. The prevalence of adult ADHD in a study in Iran was 3.8% in the general population [61]. Meanwhile, Hamzeloo et al., in a study conducted among adult male prisoners in Iran, reported a prevalence of adult ADHD among them of 16.2% [62]. They reported high comorbidity of this disorder along with other psychiatric disorders. In addition to the differences in the prevalence of this disorder in these areas, a group of experts consider ADHD to be a disorder related to cultural construct and states that part of the difference in the prevalence of this disorder in different parts of the world is due to this issue [63].

In an international study in 10 countries, Glind et al. reported an adult ADHD comorbidity with substance use disorder of 40.9% among 3,558 treatment seeking substance use disorder patients [64]. In a study of 1,205 substance use patients, Oortmerssen et al. reported a higher prevalence of bipolar disorder as a comorbid disorder in a subtype of patients with adult ADHD who were more hyperactive / impulsive [65]. Also, there is a lot of emphasis on following the best treatment method in these patients [66].

In a study of 90 patients with bipolar disorder in the euthymic phase, Karaahmet et al. reported an adult ADHD comorbidity of 23.3% in these patients [14]. This group of patients had a clear decline in academic performance compared to the group without ADHD. Also, ADHD was associated with earlier onset of mood episodes in these patients. The latter point is clearly consistent with the results of our study.

Nierenberg et al., in a study referring to the comorbidity of 60 to 90% of ADHD disorders in childhood and adolescence with bipolar disorder, described the prevalence and implication of this disorder in adulthood in bipolar patients as less clear [67] They reported lifetime prevalence of comorbid ADHD in 5.8% and 14.7% of male and female patients, respectively. They stated that the onset of mood disorders in this group of patients was 5 years earlier than other patients. They also mentioned the higher severity of the disease and the higher rate of substance use disorder in this group. Also, in a study of 103 outpatients with bipolar disorder, Tamam et al. reported a prevalence of adult ADHD comorbidity among them of 12.6% [68]. They also reported the onset of the disease at a younger age and a higher number of hospitalizations compared to patients without this comorbidity. In a study in Iran, 152 outpatients with bipolar disorder with a mean age of 33.5 years were evaluated for comorbidity with adult ADHD. 30.9% of them had a history of childhood ADHD and 11.8% had adult ADHD. Attention to treatments that can improve ADHD but are associated with a risk of worsening bipolar disorder has been emphasized by the authors [69].

In our study, which was performed on 150 hospitalized patients with bipolar disorder in an academic psychiatric hospital center in a continuous period of time, more than 70% of patients had commorbid of adult ADHD. According to the mentioned studies, patients with bipolar and ADHD comorbid disorders go through a more severe course of the disease. On the other hand, the psychiatric hospital where our study was conducted is the referral center for more complex psychiatric cases in Iran, therefore, this point may be one of the reasons for the high comorbidity rate between these two disorders in our study. Also, the use of accurate assessment tools for bipolar patients in terms of adult ADHD comorbidity and attention to the diagnosis of this disorder, can be effective in identifying this high percentage of comorbidity.

Another important point in this regard is to evaluate the persistence of ADHD symptoms from adolescence to adulthood. In a study by Jahangard et al., the prevalence of ADHD in young adult Iranian students was reported to be 13.4–16.5% and estimated to be higher than worldwide prevalence rate [43]. In their report, they described child ADHD as related to adult ADHD. Given that to our knowledge, this study is the first study performed on inpatients bipolar disorder in Iran regarding of comorbidity of adult ADHD, conducting similar studies in multicenter conditions with a larger sample size can help to better understand the extent of this comorbidity in more severe clinical conditions in patients with bipolar disorder.

Also, despite some views on the increasing prevalence of substance use disorders in the early onset of bipolar disorder [5], in our study, no statistically significant relationship was found between substance use in bipolar patients and the age of onset of the disorder. In a study with similar results, Liu et al., found no difference in the increased risk of developing substance use disorders between early-onset and adult bipolar patients [70]. This may be due to the possible secondary nature of substance use, depending on the patient's mood, especially during the early onset of symptoms of bipolar disorder. Although there are much different views on this issue [30] such a way that some have described substance use as a cause of bipolar disorder, some as self-medicate, and some as a separate disorder with similar risk factors. What is clear is that definitive conclusions in this area still need further evaluation and related studies. Other studies have reported a minimum lifetime prevalence of substance use disorder in bipolar patients of 40%, which is close to the results of our study [71]. Also, attention to cultural issues and the prevalence and pattern of substance use in this field can be considered [72]. As noted, in our study, all patients with recent substance use met the criteria for substance use disorder, even in cases in the first episode of the disorder. These results, in addition to the need to pay attention to diagnostic complexities, emphasize the need to pay attention to substance use patterns in the community at the time of the diagnostic evaluations.

Hunt et al. also described comorbidity between bipolar disorder and substance use disorder as common. They reported a comorbidity of 33% between the two disorders. They also reported the highest possible association with illicit drug use [73]. As in our study, half of the drug users used a combination of amphetamine and opium and a quarter used amphetamine alone. Wilens et al. in a ten-year follow-up of ADHD, from adolescence to young adult, reported a significant association between this disorder and substance use in both gender. They reported social and familial environmental factors as well as factors related to cognitive function had no significant effect in this regard [74]. In our study, there was no statistically significant relationship between adult ADHD in patients with bipolar disorder and substance abuse. Thus, it seems that, among the population under our study, bipolar disorder probably had the greatest impact on substance use. In fact, it seems that mood disorders have been an important factor in the high prevalence of substance use among patients with bipolar disorder in our study. However, a definite statement in this regard requires further evaluations and the use of larger sample sizes and longitudinal and multi-center studies.

Garakani et al., in a review of 113 patients with a dual diagnosis of bipolar disorder and substance use disorder in an inpatient setting with a mean age of 32.6 years, reported a 24% prevalence of ADHD among this group [75]. In their study, conducted at a private suburban academically center, they noted the effect of ADHD on affective lability in bipolar disorder independent of substance use. In our study, among 89 inpatients bipolar disorder with substance use disorders and in a situation similar to the age of the patients in the above study, the prevalence of comorbidity of adult ADHD was 65.2%, which is clearly more than the study mentioned. One reason for this discrepancy could be due to differences in the diagnostic tools used in the two studies. Also, unlike the study conducted in a private center, our study was conducted in a public university referral center. Due to these characteristics, it seems that patients with more severe symptoms have a higher presence in this center, which may be effective in increasing the prevalence. Also, according to various studies, the prevalence of substance use disorder is higher in the lower levels of socio-economic classes as well as among people with co-occurring mental health disorder [76–78].

Although comorbidity of substance use disorder in bipolar disorder is one of the highest rates of comorbidity in psychiatry, relatively limited conclusions and consensus have been reached on effective treatments for these conditions. This comorbidity can be associated with important effects on the recognition and management of bipolar disorder. Attention to integrated psychosocial interventions such as integrated group therapy (IGT) and other methods has been emphasized by some experts due to the difficulties of medication management in this group of patients along with psychopharmacological therapies and mood stabilizers such as Valproate [36, 79].

Also, due to the high comorbidity of psychiatric disorders with ADHD, including bipolar disorder, the need to detect and treat these disorders is highly emphasized. Considering the use of stimulant medication to treat ADHD manifestations, after stabilizing the mood with mood stabilizers as well as restrictions on the use of stimulant medication when accompanied by motor tics and psychotic symptoms, are other recommendations [80].

On the other hand, in many cases, not paying attention to the comorbidities mentioned in patients with bipolar disorder can be associated with negative therapeutic consequences and complex therapeutic resistance. This is an important point that can be considered by clinicians and requires full assessment of the severity of comorbid symptoms and related pharmacological and psychological treatments. This is especially important in terms of accompanying substance use disorder. In this regard, due to the limitations of related clinical trials in patients with bipolar disorder and comorbidity of substance use disorder (being in such a comorbidity status as a measure of exclusion criteria in many studies), we face a limitation in valid evidence for therapeutic interventions [81, 82]. This also highlights the need for relevant studies in research policy-making.

Also, the high prevalence of ADHD comorbidity among patients with bipolar disorder, especially in cases of high severity of symptoms, emphasizes the need for effective treatment of this disorder among these patients. Attention to effective treatment management in this field for the negative effects of some stimulants on mood and also, attention to some psychological interventions (despite the limited evidence of effectiveness) can be considered by clinicians [83].

On the other hand, therapeutic resistance in cases with ADHD diagnosis, especially at younger ages, may indicate comorbidity with bipolar disorder. This can be accompanied by a marked increase in morbidity and disease burden among these patients. The possible comorbidity of substance use disorder at a later age can also lead to exacerbation of related symptoms and related diagnostic problems. This issue should also be considered by clinicians for the need for diagnostic attention and possible changes in psychopharmacological and psychosocial interventions and the creation of the necessary substrates by administrators and policymakers.

### Limitations and strengths

One of the limitations of our study was its implementation in a hospital setting. In order to have more secure access to the information needed to complete the questionnaires and to ensure the patient history, it was necessary to ensure that the patients' psychiatric status was stable. It was also necessary to obtain information from patient caregivers in order to complete some parts of the history. Another important point was the complexity of the referral cases to the university hospital where the study was conducted. Thus, the focus of the study was on obtaining an accurate history of the patient's psychiatric illness.

Also, due to some overlapping symptoms between mania episode and ADHD, such as increased psychomotor activity, speech and distractibility, the patients' condition was monitored regularly to assess the appropriate time for the interview. In addition, for this purpose, during the study, a close relationship was established with the patients' physicians. Other available resources, such as patients' families and caregivers, were also used to obtain more accurate information for diagnostic conclusions in obscure situations. Despite the best efforts made to accurately distinguish ADHD symptoms from the remaining symptoms of the mania / mixed episode, it seems that the use of other questionnaires related to symptom identification and diagnosis of ADHD, such as the Conners' Adult ADHD Rating Scales(CAARS), can help increase diagnostic accuracy. This point can be used in designing related future studies.

Another point is that although there was no statistically significant relationship between ADHD in bipolar patients and substance use disorder, due to the limited sample size and the proximity of p value (0.07) to the cut-off point (0.05), the need for attention and continuation of studies with larger sample size and multi-center conditions are considered.

In this study, one of the main goals was to investigate the comorbidity of substance use disorder with bipolar disorder. Accordingly, we have examined this disorder in relation to the use of alcohol and other non-alcoholic substances other than tobacco in order to more accurately investigate the use of illicit drugs in this group of patients.

One of the strengths of our study was the assessment of the comorbidity of adult ADHD among complex cases with more severe courses of illness among many cases under study. This made it possible to achieve the prevalence of adult ADHD and substance use disorder comorbidity among this group of patients with bipolar disorder.

## Conclusions

In our study, a high prevalence of adult ADHD comorbidity was observed in patients with bipolar disorder during hospitalization. About two-thirds of patients with this comorbidity had a history of substance use. About half of the bipolar inpatients also had a history of substance use. However, there was no statistically significant relationship between substance use in bipolar patients and the age of onset of the disorder and there was no statistically significant relationship between adult ADHD in bipolar patients and substance use.

There was also an association between adult ADHD and the early onset of bipolar disorder. Paying attention to mood symptoms among children and adolescents with ADHD can be helpful in early detection of cases with comorbidity of bipolar disorder in these patients and provide effective and needed treatments sooner.

Also, due to the problems that exist in the identification and treatment of patients with adult ADHD worldwide, proper screening of patients with bipolar disorder is very important in terms of ADHD symptoms to achieve more effective treatment and more effective control of symptoms. In our study, there was no relationship between substance use disorder in bipolar patients and the age of onset of the disorder, which may indicate a less likely direct causal relationship between substance use disorder and the onset of bipolar disorder, especially in cases with early onset of the disorder. There was also no relationship between adult ADHD in bipolar patients and substance use disorder, which may have emphasized the important role of bipolar disorder in accompanying substance use disorder compared to ADHD. Due to the limitations of studies on adult ADHD comorbidity with bipolar disorder, especially in hospital settings, as well as the increased risk of association with substance use disorder, further multicenter studies in this area with larger sample sizes can increase awareness in this regard.

Also, the results of our study emphasize the need for clinicians to pay attention to overlapping symptoms between bipolar disorder, ADHD and substance use disorder. On the other hand, diagnostic complexity in the case of comorbidity of the above two or three disorders emphasizes the need for early detection of the disorder in childhood and adolescence and at the school level. A point that can be seriously considered by health policy makers and administrators. Also, in this regard, the necessity of training teachers, experts and even clinicians to refer cases in a timely manner to specialized levels of psychiatry is emphasized.

## Data Availability

The datasets used and/or analyzed during the current study are available from the corresponding author (Fatemeh Rahiminejad) on reasonable request.

## Abbreviations

ADHD: Attention deficit hyperactivity disorder
DSM-5: Diagnostic and Statistical Manual of Mental Disorders, 5th Edition
SCID-5: Structured Clinical Interview for Diagnostic and Statistical Manual of Mental Disorders, 5th Edition
